# Initial Ward Admission Followed by Early Respiratory Intermediate Care Transfer and In-Hospital Mortality

**DOI:** 10.3390/medsci14040470

**Published:** 2026-08-10

**Authors:** Txomin Zabala Hernández, Susana García Gutierrez, Amaia Aramburu Ojembarrena, Amaia García Loizaga, Myriam Aburto, Francisco Javier Moraza Cortes, Cristobal Esteban Gonzalez, María Gascón Pérez, María José Legarreta Olabarrieta, Ane Uranga

**Affiliations:** 1 Department of Pneumology, Biobizkaia Health Research Institute, Osakidetza Basque Health Service, Galdakao-Usansolo University Hospital, 48960 Galdakao, Spain; amaia.aramburuojembarrena@osakidetza.eus (A.A.O.); amaia.garcialoizaga@osakidetza.eus (A.G.L.); myriam.aburtobarreneche@osakidetza.eus (M.A.); franciscojavier.morazacortes@osakidetza.eus (F.J.M.C.); cristobal.est@gmail.com (C.E.G.); ane.urangaecheverria@osakidetza.eus (A.U.); 2 Biosistemak, Research Unit, Galdakao-Usansolo University Hospital, 48960 Galdakao, Spain; susana.garciagutierrez@osakidetza.eus (S.G.G.); maria.gascon@bioaraba.org (M.G.P.); mariajose.legarretaolabarrieta@bio-sistemak.eus (M.J.L.O.); 3 Research Network on Chronicity, Primary Care, and Health Promotion (RICAPPS), Carlos III Health Institute (ISCIII), 28029 Madrid, Spain

**Keywords:** respiratory intermediate care unit, emergency department, admission pathway, acute respiratory failure, non-invasive respiratory support, in-hospital mortality

## Abstract

**Background/Objectives:** Respiratory intermediate care units (RICUs) manage patients with severe respiratory disease requiring advanced monitoring and non-invasive respiratory support. Early identification of patients requiring RICU care after emergency department (ED) assessment remains challenging. This study assessed whether initial admission to a conventional hospital ward followed by early RICU transfer was associated with in-hospital mortality among patients ultimately admitted to a RICU. **Methods:** We conducted a 10-year prospective observational cohort study including consecutive patients admitted to the RICU of a tertiary hospital after ED assessment. Patients admitted directly from the ED to the RICU were classified as the direct RICU admission group. Patients initially admitted to a conventional ward and transferred to the RICU within 48 h were classified as the early ward-to-RICU transfer group. The primary outcome was all-cause in-hospital mortality. Secondary outcomes included ICU admission and hospital length of stay. Multivariable logistic regression was used to assess the association between admission pathway and in-hospital mortality. **Results:** A total of 1784 patients were included: 1285 (72%) in the direct RICU admission group and 499 (28%) in the early ward-to-RICU transfer group. In-hospital mortality was higher in the early ward-to-RICU transfer group than in the direct RICU admission group (13.43% versus 5.76%; *p* < 0.0001), as were ICU admission rates (6.01% versus 2.57%; *p* = 0.0004). In the complete-case multivariable model, early ward-to-RICU transfer remained associated with higher in-hospital mortality after adjustment for sex, ECOG performance status, APACHE II score at ED presentation, Charlson Comorbidity Index, heart failure, and interstitial lung disease (OR 2.99, 95% CI 1.96–4.56; *p* < 0.0001). **Conclusions:** Among patients ultimately admitted to a RICU after ED assessment, initial ward admission followed by early RICU transfer was associated with higher in-hospital mortality. These findings suggest that initial ward admission followed by early RICU transfer may help identify a clinically vulnerable subgroup among patients ultimately requiring respiratory intermediate care. However, because admission pathway may be influenced by evolving severity, diagnostic uncertainty, treatment decisions, and organisational factors, no causal inference can be made from this observational study.

## 1. Introduction

Respiratory Intermediate Care Units (RICUs) were first defined more than two decades ago as specialised hospital units designed to manage patients with severe respiratory disease who require advanced monitoring and non-invasive respiratory support but do not initially meet criteria for Intensive Care Unit (ICU) admission [1,2]. These units provide continuous respiratory and cardiac monitoring, together with specialised staff trained in non-invasive respiratory therapies, with the primary aim of managing acute or acute-on-chronic respiratory failure, particularly through non-invasive ventilation (NIV) [3].

NIV has demonstrated clear benefits in patients with acute hypercapnic respiratory failure, especially in exacerbations of chronic obstructive pulmonary disease (COPD), reducing the need for invasive mechanical ventilation and improving survival [4,5,6]. Over time, RICUs have expanded their scope beyond COPD to include a broader range of severe respiratory conditions, such as pneumonia, pulmonary embolism, and interstitial lung disease, while also incorporating other non-invasive respiratory support modalities, such as continuous positive airway pressure (CPAP) and high-flow nasal cannula oxygen therapy (HFNC) [7]. As a result, RICUs have become a key component in the care of patients with severe respiratory disease, acting as an intermediate level between conventional hospital wards and the ICU.

Several studies have shown that RICUs are safe and cost-effective, reducing ICU admissions without increasing mortality [8,9]. Moreover, evidence from ICUs suggests that a significant proportion of admissions may be avoidable, highlighting the importance of appropriate patient allocation and efficient use of critical care resources [10]. Despite these findings, the availability and structure of RICUs vary widely across healthcare systems, and until recently they were absent from many tertiary hospitals [11]. The SARS-CoV-2 pandemic highlighted the clinical value of these units, leading to a rapid expansion of RICUs in many countries and reinforcing their role in the management of severe respiratory disease [12,13].

Delayed admission to an appropriate level of monitored care has been associated with adverse outcomes in acute illness, particularly among patients requiring intensive care [14,15,16,17]. This association has also been reported in patients with acute respiratory failure and community-acquired pneumonia, and is generally attributed to delays in the recognition of clinical severity and in the initiation of appropriate monitoring and organ support [18].

Whether a similar association exists for patients requiring respiratory intermediate care remains unclear. Prognostic factors and organisational determinants of outcomes in RICUs remain poorly characterised [19]. In particular, no previous studies have specifically examined whether initial admission to a conventional hospital ward, followed by early transfer to the RICU, is associated with adverse outcomes among patients ultimately requiring respiratory intermediate care.

In clinical practice, early identification of patients requiring RICU care may be challenging. Patients with overt acute-on-chronic ventilatory failure, such as COPD exacerbations requiring NIV, may be more readily identified as needing specialised respiratory support. In contrast, patients with pneumonia, sepsis, pulmonary embolism, or interstitial lung disease may present with less specific or rapidly evolving signs of respiratory deterioration. These patients may initially be admitted to a conventional ward before subsequently requiring transfer to the RICU. This admission pathway may reflect diagnostic uncertainty, evolving severity, organisational factors, or delayed recognition of the need for intermediate respiratory monitoring and support.

We hypothesised that, among patients ultimately admitted to the RICU, initial admission to a conventional ward followed by early RICU transfer would be associated with worse clinical outcomes compared with direct RICU admission from the ED. The aim of this study was therefore to assess the association between initial ward admission followed by early RICU transfer and in-hospital mortality, as well as secondary outcomes including ICU admission and hospital length of stay.

## 2. Materials and Methods

**Study design and population:** A prospective observational cohort study was conducted including all consecutive patients admitted to the Respiratory Intermediate Care Unit (RICU) of Galdakao-Usansolo University Hospital over a 10-year period, from February 2007 to May 2016. The study focused on the early admission pathway of patients ultimately requiring RICU care after emergency department (ED) assessment. Patients who had been hospitalised for more than 48 h before RICU admission were excluded from the primary analysis. This threshold was selected to focus on early post-ED admission pathways and to reduce the inclusion of patients whose need for RICU admission may have been primarily related to later in-hospital clinical deterioration, hospital-acquired complications, or events occurring after the initial admission episode.

The source cohort was restricted to patients who were ultimately admitted to the RICU. Therefore, patients initially admitted to a conventional ward who recovered without RICU admission, deteriorated and were transferred directly to the ICU, died before RICU transfer, remained on the ward despite clinical deterioration, or were not considered candidates for escalation of care were not included in the study database.

**RICU characteristics and admission criteria:** Galdakao-Usansolo University Hospital is a 400-bed tertiary hospital providing care to a population of approximately 313,000 inhabitants. The RICU is an open unit integrated within the respiratory medicine department, with a maximum capacity of seven beds. The unit is staffed with a nurse-to-patient ratio of 1:4 and is supervised by a pulmonologist for 12 h per day, with shared on-call coverage between pulmonology and cardiology services during the remaining hours.

RICU admission criteria were based on European and local guidelines [20,21,22]. These criteria were intended to identify patients requiring a level of respiratory monitoring or non-invasive respiratory support beyond that available on a conventional ward, but without an immediate indication for conventional ICU admission. Final admission decisions were made on an individual basis, considering the patient’s overall clinical situation, age, comorbidity burden, baseline functional status, goals of care, and patient preferences.

Because the present study focused on patients ultimately requiring RICU care after emergency department assessment, step-down admissions from the ICU or post-anaesthesia recovery unit were not included in the analysed admission pathway groups.

The main criteria guiding RICU admission included the need for non-invasive ventilation for acute or acute-on-chronic respiratory failure; severe respiratory failure requiring non-invasive monitoring and/or high-flow nasal cannula oxygen therapy; complex thoracic trauma or pleural drainage requiring monitoring; and bronchoscopy in patients requiring non-invasive respiratory support due to clinical severity. The full institutional criteria are provided in Supplementary Section S1.

During the study period, patients admitted to conventional wards received standard medical care according to prevailing clinical guidelines and local protocols. Conventional oxygen therapy could be provided on hospital wards; however, advanced non-invasive respiratory support modalities, including non-invasive ventilation, CPAP, and high-flow nasal cannula oxygen therapy, were not routinely available on conventional wards and were mainly provided in the RICU or ICU.

**Admission pathway groups:** Patients were classified according to their initial admission pathway after ED assessment. Patients admitted directly from the ED to the RICU were classified as the direct RICU admission group. Patients initially admitted to a conventional hospital ward and subsequently transferred to the RICU within the first 48 h of hospitalisation were classified as the early ward-to-RICU transfer group.

This exposure definition was based on the observed admission pathway and should not be interpreted as an adjudicated assessment of the appropriateness of the initial ED disposition decision. No independent blinded review or retrospective application of predefined RICU eligibility criteria was performed to determine whether the initial ward admission was inappropriate in individual cases.

**Data collection:** The following variables were prospectively collected: socio-demographic characteristics; baseline clinical status, including comorbidities assessed using the Charlson Comorbidity Index [23] and functional status assessed using the Eastern Cooperative Oncology Group (ECOG) performance status score [24]; clinical and laboratory variables recorded at ED presentation and at RICU admission; and severity of illness assessed using the Acute Physiology and Chronic Health Evaluation II (APACHE II) score [25]. For the purposes of this study, APACHE II score at ED presentation was calculated using the worst available physiological and laboratory values recorded during the initial ED assessment. Although APACHE II was originally developed as a severity-of-disease classification system for critically ill patients, previous studies have evaluated APACHE-based models using data obtained in the ED for prognostic assessment of in-hospital mortality [26]. In the present study, APACHE II at ED presentation was therefore used as a standardized measure of acute physiological severity at presentation, rather than as a formally validated RICU triage tool. Primary diagnosis was assigned by the attending physician at the time of RICU admission, based on the main clinical condition considered responsible for the need for respiratory intermediate care after review of the available clinical, laboratory, and imaging information. For the purposes of the analysis, one main diagnosis was recorded for each admission.

**Outcomes:** The primary outcome was all-cause in-hospital mortality during the index hospital admission. Secondary outcomes included subsequent ICU admission and hospital length of stay. Hospital length of stay was defined as the number of days from hospital admission to hospital discharge or in-hospital death.

**Statistical analysis:** Continuous variables are expressed as mean (standard deviation) or median [interquartile range], as appropriate, and categorical variables as frequencies and percentages. Comparisons between two groups were performed using the chi-square test or Fisher’s exact test for categorical variables, as appropriate, and the Wilcoxon rank-sum test for continuous variables. Comparisons across more than two groups were performed using the chi-square test for categorical variables and the Kruskal–Wallis test for continuous variables. All statistical tests were two-sided, and a *p* value < 0.05 was considered statistically significant.

An exploratory sensitivity analysis was performed to assess whether outcomes varied according to time from hospital admission to RICU transfer. For this analysis, patients admitted to the RICU up to 72 h after hospital admission were included and classified into four admission-time categories: direct ED-to-RICU admission, ward-to-RICU transfer within 24 h, ward-to-RICU transfer between 24 and 48 h, and ward-to-RICU transfer between 48 and 72 h.

Missing data were reported for each variable included in the descriptive analyses and in the multivariable model. Percentages were calculated using available data for each variable. Variables with substantial missingness were retained in the descriptive analyses when clinically relevant, but were interpreted cautiously and were not included in the primary multivariable model. No imputation was performed.

A multivariable logistic regression model was used to assess the association between admission pathway and in-hospital mortality. Candidate covariates were selected based on clinical relevance, availability, and their potential role as confounders or prognostic factors. Univariable associations with in-hospital mortality were also considered to inform model building, but variables were not selected solely on the basis of statistical significance. The initial set of candidate covariates included age, sex, ECOG performance status, Charlson Comorbidity Index, APACHE II score at ED presentation, chronic respiratory failure, home non-invasive ventilation, primary diagnosis, PaO_2_/FiO_2_ at ED presentation, arterial pH or PaCO_2_ at ED presentation, and renal function markers. A parsimonious final model was then derived considering clinical plausibility, statistical contribution, missing data, multicollinearity, and model stability. Variables reflecting overlapping clinical constructs, such as gas exchange, acid-base status, and renal function, were considered during model building. However, they were not retained in the final model when they did not improve model stability or statistical contribution, or when their inclusion would have reduced the complete-case sample size because of missing data. The final model included admission pathway, sex, ECOG performance status, APACHE II score at ED presentation, Charlson Comorbidity Index, heart failure, and interstitial lung disease. APACHE II score and Charlson Comorbidity Index were modelled as continuous variables. ECOG performance status was modelled as a categorical variable rather than as a continuous variable because it is an ordinal clinical scale; three clinically meaningful categories were used: 0–1, 2, and 3, 4. Selected primary diagnosis indicators were included in the model to account for differences in diagnostic case mix. Because the diagnosis variable was recorded at RICU admission and may have been finalised after the initial ED disposition decision, it was interpreted as a case-mix adjustment variable rather than as a variable necessarily available to guide the initial ED triage decision. The final model was fitted using complete-case analysis. Standardized mean differences were calculated for variables included in the multivariable model in the complete-case cohort to describe between-group imbalance independently of sample size.

Multicollinearity among predictors was assessed using variance inflation factors (VIFs) or generalized variance inflation factors (GVIFs), as appropriate. Model discrimination was assessed using the area under the receiver operating characteristic curve (AUC). Overall prediction error was assessed using the Brier score. Calibration was assessed using the Hosmer–Lemeshow goodness-of-fit test, calibration intercept, and calibration slope. Internal validation was performed using stratified 5-fold cross-validation. Out-of-fold AUC and Brier score were used to assess internally validated discrimination and overall prediction error.

Statistical analyses were conducted using SAS version 9.4 for Windows (SAS Institute, Cary, NC, USA) and R version 4.1.1.

**Statement of Ethics:** The study was conducted in accordance with the Declaration of Helsinki and was approved by the local Clinical Research Ethics Committee of Galdakao-Usansolo University Hospital (protocol/reference number: 03/2007; approval date: 25 January 2007). Given the observational nature of the study and the use of routinely collected clinical data, the requirement for written informed consent was waived by the ethics committee.

## 3. Results

### 3.1. Study Population

**Figure 1:**
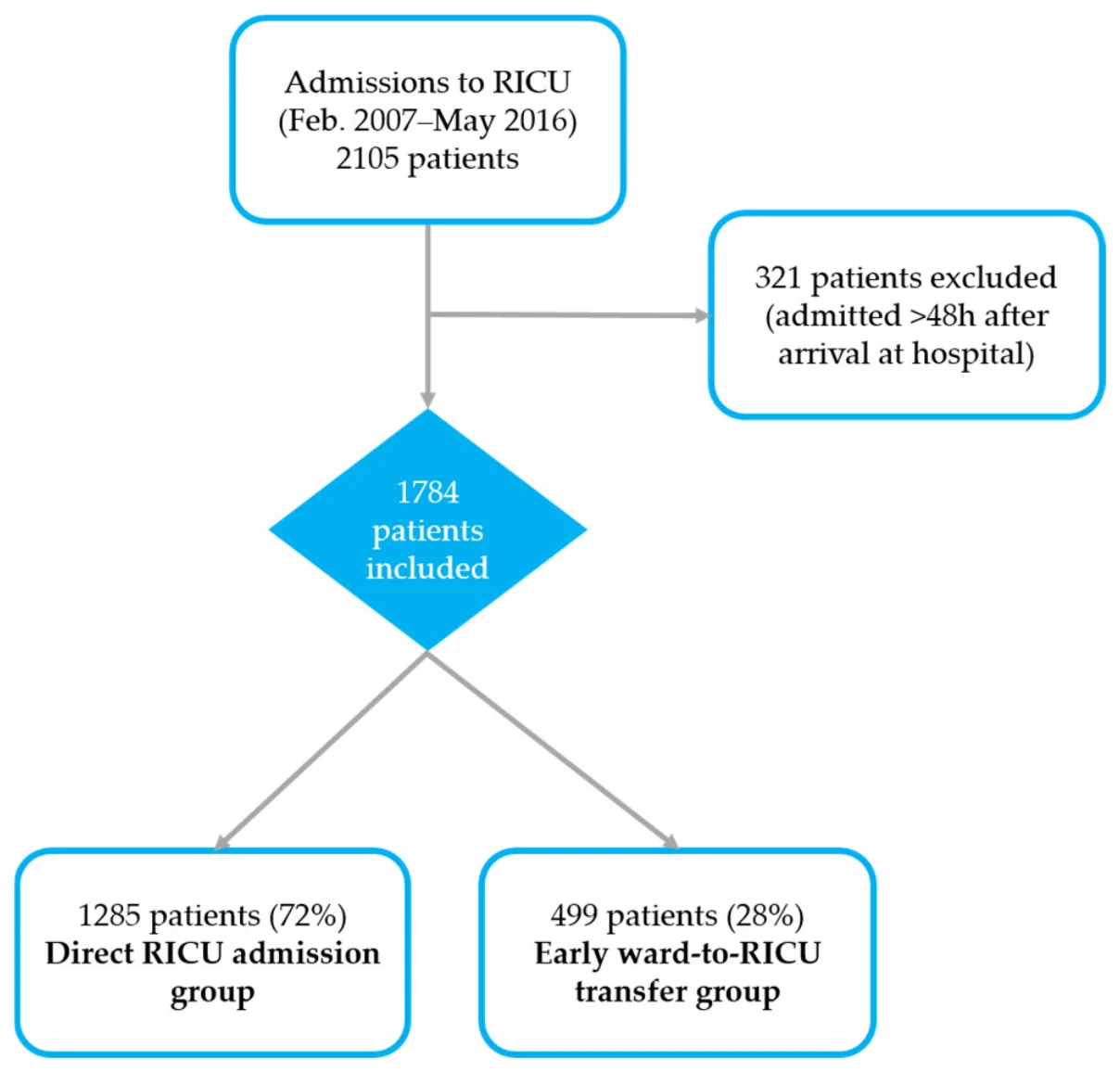
Flow chart of patients included in the study.

During the study period, a total of 2105 patients were admitted to the RICU. Of these, 321 patients had been hospitalised for more than 48 h before RICU admission and were excluded from the primary analysis, as their clinical status may have been influenced by later in-hospital evolution. The final study cohort comprised 1784 patients. Overall, 1285 patients (72.03%) were admitted directly from the ED to the RICU and were classified as the direct RICU admission group. The remaining 499 patients (27.97%) were initially admitted to a conventional hospital ward and subsequently transferred to the RICU within the first 48 h of hospitalisation, and were classified as the early ward-to-RICU transfer group (Figure 1).

Missing data for variables included in the descriptive analyses and in the multivariable model are reported in Supplementary Table S1.

### 3.2. Baseline Characteristics

Baseline socio-demographic characteristics and comorbidities are summarised in Table 1. The majority of patients were male in both groups, although the proportion of men was slightly higher in the direct RICU admission group than in the early ward-to-RICU transfer group (61.09% vs. 55.31%, *p* = 0.0256). No statistically significant differences were observed between groups in age, body mass index, baseline functional status assessed by ECOG performance status, or comorbidity burden assessed by the Charlson Comorbidity Index. Chronic respiratory failure and long-term home non-invasive ventilation were more frequent among patients directly admitted to the RICU than among those initially admitted to a conventional ward and subsequently transferred to the RICU (35.80% vs. 28.66%, *p* = 0.0042; and 17.12% vs. 10.42%, *p* = 0.0004, respectively).

### 3.3. Clinical Severity at Emergency Department Presentation

Clinical and laboratory variables recorded at ED presentation are shown in Table 2. Both groups presented with signs of acute respiratory compromise, including tachypnoea and hypoxaemia. Overall acute severity assessed by APACHE II score at ED presentation did not differ significantly between the direct RICU admission and early ward-to-RICU transfer groups (16.04 ± 7.43 vs. 15.21 ± 5.91, *p* = 0.0894).

However, patients admitted directly to the RICU showed higher arterial carbon dioxide levels and lower arterial pH at ED presentation compared with the early ward-to-RICU transfer group (arterial pCO_2_ 55.96 ± 23.35 vs. 49.57 ± 17.43 mmHg, *p* < 0.0001; and arterial pH 7.36 ± 0.10 vs. 7.39 ± 0.08, *p* < 0.0001), consistent with a greater burden of acute-on-chronic ventilatory failure in this group.

### 3.4. Diagnoses Leading to RICU Admission

Primary diagnosis differed according to admission pathway (Table 3). COPD and pulmonary embolism were more frequent among patients directly admitted to the RICU, whereas heart failure and interstitial lung disease were more frequent among patients initially admitted to a conventional ward and subsequently transferred to the RICU. Pneumonia was also numerically more frequent in the early ward-to-RICU transfer group, although this difference did not reach statistical significance.

### 3.5. Clinical Outcomes

Clinical outcomes are shown in Table 4. In-hospital mortality was higher in the early ward-to-RICU transfer group than in the direct RICU admission group (13.43% vs. 5.76%, *p* < 0.0001), corresponding to an observed absolute difference of 7.67 percentage points. As described above, APACHE II score at ED presentation did not differ significantly between groups.

Patients in the early ward-to-RICU transfer group also had a higher rate of subsequent ICU admission (6.01% vs. 2.57%, *p* = 0.0004). Hospital length of stay was also longer in the early ward-to-RICU transfer group (6 [4–9] vs. 6 [4–8] days, *p* = 0.0004), whereas median RICU length of stay was 3 [2–5] days in both groups, although the distribution differed statistically (*p* = 0.0392). These secondary outcomes should be interpreted descriptively.

### 3.6. Exploratory Analysis According to Admission Period

An exploratory analysis was performed according to three predefined admission periods: 2007–2009, 2010–2012, and 2013–2016. The distribution of admission pathway groups remained stable across periods. The proportion of patients initially admitted to a conventional ward and subsequently transferred to the RICU within 48 h was 28.38% in 2007–2009, 27.93% in 2010–2012, and 27.72% in 2013–2016 (*p* = 0.9678).

However, temporal differences were observed in patient characteristics, severity, diagnostic case mix, and outcomes. The Charlson Comorbidity Index decreased over time, from a median of 2 [1–3] in the first two periods to 1 [0–2] in 2013–2016 (*p* < 0.0001). APACHE II score at ED presentation also decreased across periods, from 16.83 (7.22) in 2007–2009 to 14.89 (7.19) in 2013–2016 (*p* < 0.0001), as did APACHE II score at RICU admission. Overall, in-hospital mortality decreased over time, from 9.78% and 9.12% in the first two periods to 5.71% in 2013–2016 (*p* = 0.0145). The full analysis is shown in Supplementary Table S3.

### 3.7. Exploratory Analysis According to Time to RICU Admission

To further explore the clinical relevance of the 48 h threshold used in the primary analysis, an exploratory analysis was performed including patients admitted to the RICU within 72 h of hospital admission. Patients were classified into four admission-time categories: direct ED-to-RICU admission, ward-to-RICU transfer within 24 h, ward-to-RICU transfer between 24 and 48 h, and ward-to-RICU transfer between 48 and 72 h.

In-hospital mortality increased progressively across these four categories: 5.76% among patients admitted directly from the ED to the RICU, 11.23% among those transferred within 24 h, 20.69% among those transferred between 24 and 48 h, and 33.33% among those transferred between 48 and 72 h (*p* < 0.0001). A similar gradient was observed for subsequent ICU admission, which occurred in 2.57%, 5.48%, 7.76%, and 8.89% of patients, respectively (*p* = 0.0007).

The full exploratory analysis is shown in Supplementary Table S2A–D. This analysis should be interpreted as descriptive and hypothesis-generating, rather than as evidence of a causal effect of transfer time itself.

### 3.8. Multivariable Analysis of Factors Associated with In-Hospital Mortality

In the complete-case multivariable logistic regression model (N = 1502) (Table 5), early ward-to-RICU transfer remained associated with higher in-hospital mortality compared with direct RICU admission after adjustment for sex, ECOG performance status, APACHE II score at ED presentation, Charlson Comorbidity Index, heart failure, and interstitial lung disease (OR 2.99, 95% CI 1.96–4.56; *p* < 0.0001).

Other variables associated with in-hospital mortality were female sex (OR 1.56, 95% CI 1.01–2.43; *p* = 0.0461), ECOG performance status 2 (OR 1.93, 95% CI 1.10–3.39; *p* = 0.0217), ECOG performance status 3–4 (OR 3.15, 95% CI 1.76–5.65; *p* = 0.0001), APACHE II score at ED presentation (OR 1.05 per point, 95% CI 1.02–1.08; *p* = 0.0014), Charlson Comorbidity Index (OR 1.16 per point, 95% CI 1.03–1.32; *p* = 0.0180), heart failure (OR 1.97, 95% CI 1.16–3.33; *p* = 0.0118), and interstitial lung disease (OR 6.48, 95% CI 2.78–15.11; *p* < 0.0001).

No relevant multicollinearity was observed among the predictors included in the model; adjusted GVIF values ranged from 1.02 to 1.06. The model showed acceptable discrimination, with an AUC of 0.769 (95% CI 0.72–0.81), and a Brier score of 0.0611. The calibration intercept was −0.209 (95% CI −0.69 to 0.28), and the calibration slope was 0.903 (95% CI 0.70 to 1.10). However, the Hosmer–Lemeshow test was statistically significant (*p* = 0.017), indicating imperfect calibration across risk groups.

In stratified 5-fold cross-validation, the model showed moderate internally validated discrimination, with an out-of-fold AUC of 0.745 (95% CI 0.698–0.791) and a Brier score of 0.0624.

## 4. Discussion

In this large observational cohort of patients ultimately admitted to a RICU after ED assessment, initial admission to a conventional ward followed by transfer to the RICU within 48 h was associated with significantly higher in-hospital mortality compared with direct RICU admission from the ED. This association remained present after adjustment for clinically relevant covariates, including sex, baseline functional status, severity at ED presentation, comorbidity burden, and selected primary diagnoses. These findings suggest that early ward-to-RICU transfer identifies a subgroup of patients at increased risk of adverse outcomes, but should not be interpreted as evidence that the initial ward admission was inappropriate in individual cases or that the admission pathway itself caused the observed mortality difference.

The multivariable model showed acceptable discrimination, with an AUC of 0.769 (95% CI 0.72–0.81), and a low Brier score, suggesting low overall prediction error. However, the significant Hosmer–Lemeshow test indicates imperfect calibration across risk groups. Therefore, the model should be interpreted primarily as an explanatory association model rather than as a standalone clinical prediction tool.

Importantly, patients initially admitted to a conventional ward did not present with greater overall severity at ED presentation, as assessed by APACHE II score. This finding suggests that the excess mortality observed in the early ward-to-RICU transfer group was not fully captured by global severity at ED presentation. Several explanations may account for this observation, including evolving disease severity, diagnostic uncertainty, differences in clinical phenotype, unmeasured early treatments, organisational factors, or delayed recognition of the need for specialised respiratory monitoring and support.

The exploratory analysis according to time from hospital admission to RICU admission provides additional context for the 48 h threshold used in the primary analysis. A progressive increase in in-hospital mortality and subsequent ICU admission was observed across the four admission-time categories, from direct ED-to-RICU admission to ward-to-RICU transfer within 24 h, between 24 and 48 h, and between 48 and 72 h. These findings suggest that patients initially admitted to a conventional ward and subsequently transferred to the RICU represent a progressively higher-risk subgroup, particularly when transfer occurs later after hospital admission. However, this analysis should be interpreted cautiously. The observed gradient may reflect evolving disease severity, diagnostic uncertainty, response to initial treatment, unmeasured clinical deterioration, selection effects, or organisational factors, rather than the effect of time to RICU transfer itself.

Overall, these results suggest that initial ward admission followed by early RICU transfer may represent a potentially modifiable marker of vulnerability within the acute respiratory care pathway. Previous ED studies have used the concept of undertriage to describe patients initially allocated to a lower level of care than ultimately required, and have shown that this phenomenon may be associated with delays in diagnostic and therapeutic interventions and worse outcomes [27,28]. However, in the present study we did not assess whether the initial ED disposition decision was appropriate in individual cases, because no independent review of that decision was performed. Therefore, initial ward admission followed by early RICU transfer should be interpreted as an observed care pathway associated with higher risk, rather than as a confirmed triage error.

In septic shock, initial ward admission followed by delayed ICU transfer has been associated with clinically important delays in antibiotic administration, vasopressor initiation, and ICU admission [29,30]. Although patients requiring RICU care differ from septic shock populations, these findings support the broader concept that initial allocation to a lower-intensity care setting may be associated with delayed access to specialised monitoring or organ support in selected acute conditions.

Heart failure was more frequent among patients initially admitted to a conventional ward and subsequently transferred to the RICU, and was also associated with in-hospital mortality in the multivariable model. This finding suggests that patients with acute heart failure and a prominent respiratory component may represent a clinically challenging phenotype at ED presentation. In some cases, the need for specialised respiratory monitoring or non-invasive respiratory support may not be immediately apparent. This is clinically relevant, as non-invasive respiratory support, including CPAP and NIV, has been shown to improve symptoms and clinical outcomes in patients with acute cardiogenic pulmonary oedema [31].

Interstitial lung disease was also associated with in-hospital mortality in the multivariable model. This is consistent with the high-risk nature of acute respiratory deterioration in patients with interstitial lung disease, in whom hypoxaemic respiratory failure may progress rapidly and may be difficult to reverse once established. Although pneumonia was numerically more frequent in the early ward-to-RICU transfer group, this difference did not reach statistical significance. Nevertheless, pneumonia remains a clinically relevant scenario because respiratory deterioration can be rapid and difficult to predict, and previous studies have shown that delayed admission to higher levels of care in patients with community-acquired pneumonia is associated with worse outcomes [32].

Several factors may explain why some patients requiring RICU care were initially admitted to a conventional ward. First, unlike other areas of acute care, there is no widely adopted RICU-specific triage tool to identify, at ED presentation, patients with severe respiratory disease who may benefit from continuous monitoring and early non-invasive respiratory support [8,33]. Second, organisational factors may also have contributed. Bed availability and unit capacity are known to influence admission decisions in ICU settings [34], and similar mechanisms may plausibly operate in RICUs. Finally, recognition of the need for RICU admission may differ according to the clinical phenotype. In our cohort, patients with chronic respiratory failure, long-term home NIV, hypercapnia, and lower arterial pH were more frequently admitted directly to the RICU, suggesting that acute-on-chronic ventilatory failure was more readily identified as requiring specialised respiratory care. This may reflect the historical role of NIV as a core therapy in RICUs, whereas patients with predominantly hypoxaemic or less overt respiratory presentations may be more likely to follow an initial ward admission pathway before requiring RICU transfer.

Although causality cannot be inferred from the present study, several mechanisms may plausibly explain the association between initial ward admission followed by early RICU transfer and adverse outcomes. RICUs provide closer monitoring, specialised respiratory expertise, and higher nurse staffing than conventional wards. Previous studies in ICU settings have shown that delayed admission to higher levels of care is associated with worse outcomes, including higher mortality and longer hospital stay [15,35]. In addition, higher nurse staffing and lower patient-to-nurse ratios have been associated with earlier detection of clinical deterioration and better outcomes in critically ill patients [36,37], and continuous non-invasive monitoring in hospitalised patients has been associated with reduced mortality [38]. In this context, initial admission to a conventional ward may reduce opportunities for early recognition and treatment of worsening respiratory failure in selected patients, although this mechanism could not be directly assessed in our study.

Our findings are also consistent with previous research supporting the clinical role of RICUs. Confalonieri et al. reported lower in-hospital mortality, reduced ICU admission rates, and shorter hospital stays among patients managed in RICUs compared with those treated in conventional wards or in the ED [39]. Beyond clinical outcomes, increasing evidence also supports the cost-effectiveness of these units. Heili-Frades et al. showed that the implementation of an RICU could substantially reduce healthcare costs by decreasing the need for ICU admissions and shortening ICU length of stay [8]. Taken together, these data support the role of RICUs as an intermediate level of care for selected patients with severe respiratory disease.

These results have several potential implications for clinical practice. First, they highlight the importance of improving early identification of patients who may require specialised respiratory monitoring and non-invasive respiratory support. Structured ED-to-RICU pathways, explicit admission criteria, and systematic reassessment of patients admitted to conventional wards may help identify high-risk patients more reliably, particularly in conditions with heterogeneous clinical presentation, such as heart failure, pneumonia, or interstitial lung disease. Second, ED and ward protocols should emphasise early recognition of respiratory deterioration by integrating clinical signs, gas exchange abnormalities, comorbidities, baseline functional status, and response to initial treatment. Finally, these findings support the continued development and optimisation of RICUs as intermediate care facilities capable of providing timely specialised respiratory support while reducing pressure on intensive care units.

This study has several strengths. First, it includes a large prospective cohort of consecutive patients admitted to a RICU over a 10-year period, reflecting real-world respiratory intermediate care practice. Second, detailed clinical, functional, physiological, and laboratory data were collected at two clinically relevant time points: ED presentation and RICU admission. This allowed assessment of both baseline severity at presentation and clinical status at the time of respiratory intermediate care admission. Third, the exposure was based on a clearly defined and clinically meaningful admission pathway: direct RICU admission from the ED versus initial conventional ward admission followed by early RICU transfer. Patients hospitalised for more than 48 h before RICU admission were excluded from the primary analysis to reduce the influence of later in-hospital deterioration, hospital-acquired complications, or events unrelated to the initial admission episode. Finally, the analysis included assessment of missing data, standardized mean differences in the complete-case cohort, evaluation of multicollinearity, model discrimination and calibration, internal validation by cross-validation, and exploratory analyses according to admission period and time to RICU admission.

Several limitations should also be acknowledged. First, the cohort was restricted to patients who were ultimately admitted to the RICU. As a result, the study does not represent all patients assessed in the ED with acute respiratory deterioration, nor all patients initially admitted to conventional wards. Patients who improved without RICU admission, died before transfer, were transferred directly to the ICU, remained on the ward despite deterioration, or were not considered candidates for escalation of care were not captured. This selection process may have introduced selection bias. In addition, classification in the early ward-to-RICU transfer group required patients to survive until RICU admission, which may have introduced survivor-selection or immortal-time bias. Therefore, the findings should be interpreted as associations among patients eventually admitted to the RICU and should not be extrapolated to all patients assessed in the ED or admitted to conventional wards.

Second, although patients were managed according to prevailing clinical guidelines and local protocols, detailed information on treatments administered before RICU admission was not systematically available. These included oxygen flow, timing of antibiotic therapy, diuretic therapy, bronchodilator treatment, monitoring intensity, and any exceptional use of non-invasive respiratory support outside the RICU. Therefore, we could not determine whether differences in pre-RICU management contributed to the observed association between early ward-to-RICU transfer and in-hospital mortality. In addition, precise time intervals between ED presentation, ward admission, clinical deterioration, and RICU admission were not available. Consequently, the exploratory analysis according to time to RICU admission should be interpreted as descriptive and hypothesis-generating, and does not replace a formal time-dependent model.

Third, the multivariable model was developed for explanatory purposes and was not intended or validated as a standalone clinical prediction tool. Although the model showed acceptable discrimination and low overall prediction error, calibration was imperfect according to the Hosmer–Lemeshow test. Residual confounding and confounding by indication cannot be excluded, despite adjustment for clinically relevant covariates. Secondary outcomes should also be interpreted cautiously, as ICU admission may have been influenced by treatment limitations, clinical judgement, ICU bed availability, and goals of care, while RICU and hospital length of stay may have been affected by the competing risk of in-hospital death and by the skewed distribution of time-to-discharge outcomes. We also did not perform formal interaction or stratified analyses according to primary diagnosis; therefore, diagnosis-specific differences should be interpreted as descriptive and hypothesis-generating.

Finally, the study was conducted in a single centre over a long period, from 2007 to 2016, during which clinical practice, respiratory support strategies, ED pathways, and RICU organisation may have changed. Although the proportion of patients initially admitted to a conventional ward and subsequently transferred to the RICU remained stable across admission periods, patient characteristics, severity of illness, diagnostic case mix, and in-hospital mortality varied over time. Because admission period was not included in the final multivariable model, residual confounding related to secular changes cannot be excluded. In addition, patient inclusion ended in 2016; therefore, changes in respiratory care pathways over time may limit direct applicability to current practice. However, the cohort represents a pre-COVID-19 population and may still reflect a clinical context comparable to current non-pandemic respiratory care settings.

## 5. Conclusions

In this prospective observational cohort of patients ultimately admitted to a RICU after ED assessment, initial admission to a conventional ward followed by early RICU transfer was associated with higher in-hospital mortality compared with direct RICU admission from the ED. An exploratory analysis according to time to RICU admission showed a progressive increase in mortality across admission-time categories, suggesting that this admission pathway identifies a clinically vulnerable subgroup among patients requiring respiratory intermediate care.

These findings should not be interpreted as evidence that the admission pathway itself caused the observed mortality difference. Rather, they highlight the need to improve early recognition and reassessment pathways for patients with acute respiratory deterioration who may benefit from specialised respiratory monitoring and non-invasive respiratory support.

## Figures and Tables

**Table 1:** Baseline socio-demographic characteristics and comorbidities according to admission pathway.

	Total (N = 1784)	Direct RICU Admission Group (N = 1285)	Early Ward-to-RICU Transfer Group (N = 499)	*p*
Socio-demographic variables				
Sex (male)	1061 (59.47)	785 (61.09)	276 (55.31)	0.0256 *
Age (years)	69.25 (14.72)	68.89 (14.79)	70.16 (14.49)	0.0704
Height (m)	1.63 (0.10)	1.63 (0.09)	1.63 (0.10)	0.4456
Weight (kg)	78.67 (22.28)	79.11 (21.91)	77.50 (23.22)	0.0719
BMI	29.63 (7.98)	29.76 (7.92)	29.29 (8.12)	0.1792
Baseline situation				
ECOG				0.7378
0, 1	783 (44.74)	572 (45.22)	211 (43.51)	
2	593 (33.89)	422 (33.36)	171 (35.26)	
3, 4	374 (21.37)	271 (21.42)	103 (21.24)	
Baseline dyspnoea (mMRC) > 1	923 (56.04)	665 (55.74)	258 (56.83)	0.6914
Chronic NIV	272 (15.25)	220 (17.12)	52 (10.42)	0.0004 *
Home CPAP	51 (2.86)	41 (3.19)	10 (2.00)	0.1770
Chronic respiratory failure	603 (33.80)	460 (35.80)	143 (28.66)	0.0042 *
Comorbidities				
Cardiovascular disease	836 (46.86)	588 (45.76)	248 (49.70)	0.1344
Chronic pulmonary disease	984 (55.16)	724 (56.34)	260 (52.10)	0.1062
Ulcerative disease	116 (6.50)	83 (6.46)	33 (6.61)	0.9057
Liver disease	88 (4.94)	70 (5.45)	18 (3.61)	0.2688
Nephrological disease	97 (5.44)	72 (5.60)	25 (5.01)	0.1939
Diabetes	427 (23.93)	313 (24.36)	114 (22.84)	0.3300
Cancer	137 (7.68)	99 (7.70)	38 (7.62)	0.9494
Dementia	62 (3.48)	47 (3.66)	15 (3.01)	0.5000
Cerebrovascular disease	181 (10.15)	125 (9.73)	56 (11.22)	0.3479
Connective tissue disease	55 (3.08)	41 (3.19)	14 (2.81)	0.6728
Hemiplegia	46 (2.58)	32 (2.49)	14 (2.81)	0.7060
AIDS	2 (0.11)	1 (0.08)	1 (0.20)	0.4813
Arterial hypertension	1032 (57.85)	744 (57.90)	288 (57.72)	0.9439
Charlson Index	2 [1–3]	2 [1–3]	1 [1–3]	0.4611
Charlson Index > 2	475 (26.63)	350 (27.24)	125 (25.05)	0.3482

Data are presented as *n* (%) unless otherwise stated. Continuous variables are presented as mean (standard deviation), except for the Charlson Comorbidity Index, which is presented as median [interquartile range]. Percentages were calculated using available data for each variable. Missing data are reported in Supplementary Table S1. * *p* < 0.05. BMI: body mass index; CPAP: continuous positive airway pressure; ECOG: Eastern Cooperative Oncology Group performance status; mMRC: modified Medical Research Council dyspnoea scale; NIV: non-invasive ventilation; RICU: respiratory intermediate care unit; AIDS: acquired immunodeficiency syndrome.

**Table 2:** Clinical and laboratory variables at ED presentation and RICU admission.

	Total (N = 1784)	Direct RICU Admission Group (N = 1285)	Early Ward-to-RICU Transfer Group (N = 499)	*p*
Clinical variables in the ED				
Temperature (°C)	36.85 (0.81)	36.82 (0.81)	36.92 (0.83)	0.0248 *
HR (bpm)	100.25 (23.46)	100.94 (23.62)	98.46 (22.96)	0.1206
RR (rpm)	22.67 (8.19)	22.81 (8.29)	22.34 (7.96)	0.3413
Mean arterial blood pressure (mmHg)	95.12 (20.64)	96.15 (21.11)	92.47 (19.18)	0.0013 *
Glasgow	14.78 (1.09)	14.77 (1.09)	14.79 (1.09)	0.1743
APACHE II score in the ED	15.81 (7.06)	16.04 (7.43)	15.21 (5.91)	0.0894
Laboratory variables in the ED				
arterial pCO_2_ (mmHg)	54.19 (22.05)	55.96 (23.35)	49.57 (17.43)	<0.0001 *
arterial pO_2_ (mmHg)	59.54 (22.19)	60.33 (23.76)	57.45 (17.16)	0.1296
O_2_ saturation (%)	84.65 (11.48)	84.41 (11.85)	85.25 (10.45)	0.7540
PaO_2_/FiO_2_ (mmHg)	242.87 (91.65)	241.62 (94.60)	246.28 (83.09)	0.1617
Arterial pH	7.37 (0.09)	7.36 (0.10)	7.39 (0.08)	<0.0001 *
Leukocytes (×10^3^/mL)	11.99 (5.65)	12.14 (5.64)	11.62 (5.69)	0.0233 *
Platelets (×10^3^/mL)	233.97 (99.38)	236.41 (101.68)	227.67 (92.97)	0.0499 *
Haematocrit (%)	41.49 (6.62)	41.85 (6.61)	40.58 (6.59)	<0.0001 *
Creatinine (mg/dL)	1.11 (0.71)	1.09 (0.68)	1.14 (0.78)	0.5681
Urea (mg/dL)	57.28 (37.53)	56.42 (37.39)	59.48 (37.84)	0.3625
Serum Na+ (mEq/L)	138.47 (4.51)	138.66 (4.31)	137.98 (4.97)	0.0070 *
Serum K+ (mEq/L)	4.58 (0.71)	4.61 (0.71)	4.52 (0.70)	0.0222 *
Glucose (mg/dL)	155.81 (87.05)	157.65 (93.49)	151.02 (67.41)	0.2976
Bilirubin (mg/dL)	0.69 (0.53)	0.68 (0.52)	0.69 (0.56)	0.5603
Albumin (g/dL)	3.83 (0.55)	3.85 (0.52)	3.76 (0.62)	0.0536
Prothrombin activity (%)	79.58 (26.19)	80.59 (25.63)	76.80 (27.51)	0.0045 *
Clinical variables on admission to the RICU				
HR (bpm)	91.78 (19.74)	91.05 (19.36)	93.67 (20.58)	0.0147 *
RR (rpm)	22.78 (6.34)	22.41 (6.20)	23.74 (6.60)	0.0004 *
SBP (mmHg)	129.12 (23.68)	129.61 (23.39)	127.84 (24.37)	0.1203
DBP (mmHg)	71.70 (15.59)	72.42 (15.48)	69.85 (15.72)	0.0008 *
Glasgow	14.69 (1.29)	14.75 (1.09)	14.55 (1.70)	0.0154 *
APACHE II score on admission	12.20 (5.64)	11.89 (5.48)	13.04 (6.01)	0.0010 *
Laboratory variables on admission to the RICU				
Arterial pH	7.39 (0.08)	7.38 (0.07)	7.38 (0.09)	0.1181
arterial pCO_2_ (mmHg)	51.91 (19.05)	51.12 (18.41)	53.96 (20.51)	0.0350 *
arterial pO_2_ (mmHg)	73.27 (28.17)	74.77 (30.58)	69.39 (20.12)	0.0018 *
PaO_2_/FiO_2_ (mmHg)	231.85 (92.28)	235.86 (93.91)	221.37 (87.08)	0.0085 *
Glucose (mg/dL)	158.85 (68.46)	154.98 (63.67)	168.87 (78.78)	0.0014 *
Albumin (g/dL)	3.78 (0.68)	3.80 (0.56)	3.72 (0.58)	0.0024 *
Haematocrit (%)	39.79 (6.78)	40.07 (6.78)	39.05 (6.73)	0.0016 *

Data are presented as mean (standard deviation). Percentages were calculated using available data for each variable. Missing data are reported in Supplementary Table S1. * *p* < 0.05. APACHE II: Acute Physiology and Chronic Health Evaluation II; DBP: diastolic blood pressure; ED: emergency department; HR: heart rate; PaO_2_/FiO_2_: ratio of arterial oxygen partial pressure to fraction of inspired oxygen; RICU: respiratory intermediate care unit; RR: respiratory rate; SBP: systolic blood pressure.

**Table 3:** Primary diagnosis according to admission pathway.

	Total (N = 1784)	Direct RICU Admission Group (N = 1285)	Early Ward-to-RICU Transfer Group (N = 499)	*p*
COPD	403 (22.59)	318 (24.75)	85 (17.03)	0.0005 *
OHS	155 (8.69)	110 (8.56)	45 (9.02)	0.7580
Heart failure	207 (11.60)	134 (10.43)	73 (14.63)	0.0129 *
Pneumonia	363 (20.35)	248 (19.30)	115 (23.05)	0.0777
Pulmonary embolism	328 (18.39)	257 (20.00)	71 (14.23)	0.0047 *
Asthma	85 (4.76)	61 (4.75)	24 (4.81)	0.9556
ILD	35 (1.96)	20 (1.56)	15 (3.01)	0.0475 *
Neuromuscular disease	26 (1.46)	17 (1.32)	9 (1.80)	0.4470
Other diagnoses	182 (10.20)	120 (9.34)	62 (12.42)	0.0532

Data are presented as *n* (%). Percentages were calculated using the total number of patients in each admission pathway group as the denominator. For each diagnostic category, the *p* value refers to the comparison between patients with that diagnosis and patients with all other primary diagnoses. * *p* < 0.05. COPD: chronic obstructive pulmonary disease; ILD: interstitial lung disease; OHS: obesity hypoventilation syndrome; RICU: respiratory intermediate care unit. The “Other diagnoses” category included other restrictive respiratory disorders and less frequent respiratory conditions not captured by the predefined diagnostic categories.

**Table 4:** Clinical outcomes according to admission pathway.

	Total (N = 1784)	Direct RICU Admission Group (N = 1285)	Early Ward-to-RICU Transfer Group (N = 499)	*p*
ICU admission	63 (3.53)	33 (2.57)	30 (6.01)	0.0004 *
RICU length of stay (days)	3 [2–5]	3 [2–5]	3 [2–5]	0.0392 *
Hospital length of stay (days)	6 [4–8]	6 [4–8]	6 [4–9]	0.0004 *
In-hospital mortality	141 (7.90)	74 (5.76)	67 (13.43)	<0.0001 *

Data are presented as *n* (%) unless otherwise stated. Length-of-stay variables are presented as median [interquartile range]. * *p* < 0.05. ICU: intensive care unit; RICU: respiratory intermediate care unit.

**Table 5:** Multivariable logistic regression analysis of factors associated with in-hospital mortality (N = 1502).

Variables	β (SE)	OR (95% CI)	*p*
Admission pathway			
Direct RICU admission	Ref.	Ref.	
Early ward-to-RICU transfer	1.10 (0.22)	2.99 (1.96–4.56)	<0.0001 *
Sex			
Male	Ref.	Ref.	
Female	0.45 (0.22)	1.56 (1.01–2.43)	0.0461 *
ECOG			
0, 1	Ref.	Ref.	
2	0.66 (0.29)	1.93 (1.10–3.39)	0.0217 *
3, 4	1.15 (0.30)	3.15 (1.76–5.65)	0.0001 *
APACHE II, Emergency Department	0.05 (0.02)	1.05 (1.02–1.08)	0.0014 *
Charlson index	0.15 (0.06)	1.16 (1.03–1.32)	0.0180 *
Diagnostics (Binary variables)			
Heart failure	0.68 (0.27)	1.97 (1.16–3.33)	0.0118 *
ILD	1.87 (0.43)	6.48 (2.78–15.11)	<0.0001 *
AUC (95% CI)	0.769 (0.72–0.81)		
Hosmer–Lemeshow test, *p* value	0.017		
Brier score	0.0611		
Calibration intercept (95% CI)	−0.209 (−0.69 to 0.28)		
Calibration slope (95% CI)	0.903 (0.70 to 1.10)		
Stratified 5-fold cross-validated AUC (95% CI)	0.745 (0.698–0.791)		
Stratified 5-fold cross-validated Brier score	0.0624		

Data are presented as β coefficient (standard error), odds ratio (OR), 95% confidence interval (CI), and *p* value. The model included 1502 patients with complete data. * *p* < 0.05. APACHE II score at emergency department presentation and Charlson Comorbidity Index were modelled as continuous variables. ECOG performance status was modelled as a categorical variable using ECOG 0–1 as the reference category. Primary diagnosis was assigned by the attending physician at RICU admission. Heart failure and interstitial lung disease were included as binary diagnostic indicators, with all other diagnoses as the reference category. Multicollinearity was assessed using variance inflation factors or generalized variance inflation factors, as appropriate. Internal validation was performed using stratified 5-fold cross-validation. APACHE II: Acute Physiology and Chronic Health Evaluation II; AUC: area under the receiver operating characteristic curve; CI: confidence interval; ECOG: Eastern Cooperative Oncology Group performance status; ILD: interstitial lung disease; OR: odds ratio; RICU: respiratory intermediate care unit.

## Data Availability

The data that support the findings of this study are not publicly available due to privacy and ethical restrictions. Data may be available from the corresponding author upon reasonable request and subject to approval by the relevant ethics committee.

## Supplementary Materials

The following supporting information can be downloaded at: https://www.mdpi.com/article/10.3390/medsci14040470/s1, Supplementary Section S1: Institutional criteria guiding admission to the Respiratory Intermediate Care Unit; Table S1: Missing data in the primary study cohort (N=1784); Table S2A: Baseline socio-demographic characteristics and comorbidities according to time to RICU admission; Table S2B: Clinical and laboratory variables according to time to RICU admission; Table S2C: Primary diagnosis according to time to RICU admission; Table S2D: Clinical outcomes according to time to RICU admission; Table S3: Comparative analysis of the primary study cohort according to admission period (N = 1784); Table S4: Standardized mean differences for variables included in the multivariable model in the complete-case cohort.

## Abbreviations

The following abbreviations are used in this manuscript:

AIDS	acquired immunodeficiency syndrome
APACHE II	Acute Physiology and Chronic Health Evaluation II
AUC	area under the receiver operating characteristic curve
BMI	body mass index
CI	confidence interval
COPD	chronic obstructive pulmonary disease
CPAP	continuous positive airway pressure
DBP	diastolic blood pressure
ED	emergency department
ECOG	Eastern Cooperative Oncology Group
GVIF	generalized variance inflation factor
HFNC	high-flow nasal cannula oxygen therapy
HR	heart rate
ICU	intensive care unit
ILD	interstitial lung disease
mMRC	modified Medical Research Council
NIV	non-invasive ventilation
OHS	obesity hypoventilation syndrome
OR	odds ratio
PaO_2_/FiO_2_	ratio of arterial oxygen partial pressure to fraction of inspired oxygen
RICU	Respiratory Intermediate Care Unit
RR	respiratory rate
SBP	systolic blood pressure
SE	standard error
SMD	standardized mean difference
VIF	variance inflation factor
