# Supplementary material for: Initial Ward Admission Followed by Early Respiratory Intermediate Care Transfer and In-Hospital Mortality
Source: Med Sci (Basel). 2026 Aug 10;14(4):470. doi: 10.3390/medsci14040470 (PMC13515314; doi:10.3390/medsci14040470)
Supplement: Supplementary file 1 [file medsci-14-00470-s001.zip › medsci-4445517-supplementary.pdf]

Supplementary Materials: Initial Ward Admission Followed by Early Respiratory Intermediate Care Transfer and In-Hospital Mortality

Txomin Zabala Hernández, Susana García Gutierrez, Amaia Aramburu Ojembarrena, Amaia García Loizaga, Myriam Aburto Barrenechea, Francisco Javier Moraza Cortes, Cristobal Esteban Gonzalez, María Gascón Peréz, María José Legarreta Olabarrieta, and Ane Uranga

Supplementary Section S1. Institutional criteria guiding admission to the Respiratory Intermediate Care Unit

The Respiratory Intermediate Care Unit (RICU) of Galdakao-Usansolo University Hospital had predefined local criteria to guide admission. Final admission decisions were made on an individual basis, taking into account the patient's overall clinical condition, age, comorbidities, baseline functional status, goals of care, and patient preferences.

The institutional criteria guiding RICU admission were as follows:

1. Need for non-invasive ventilation for the treatment of acute respiratory failure or acute-on-chronic respiratory failure.

2. Severe respiratory failure not requiring ventilatory support but requiring non-invasive monitoring and/or high-flow nasal cannula oxygen therapy.

3. Step-down transfer from the ICU or post-anaesthesia recovery unit of stabilised patients who remained dependent on mechanical ventilation after a failed weaning attempt, either through non-invasive ventilation or tracheostomy, with the aim of progressing weaning and/or planning long-term home mechanical ventilation.

This admission pathway was part of the institutional RICU criteria but was not included in the present analysis, which focused on patients admitted after emergency department assessment.

4. Patients with complex thoracic trauma or pleural drainage requiring close monitoring.

5. Patients requiring bronchoscopy who, due to clinical severity, needed non-invasive respiratory support during the procedure.

Supplementary Table S1. Missing data in the primary study cohort (N=1784).

Variable	Missing N (%)
Socio-demographic variables	
Sex (male)	0 (0)
Age (years)	0 (0)
Height (m)	193 (10.82)
Weight (kg)	177 (9.92)
BMI	201 (11.27)
Baseline situation	
ECOG	34 (1.91)
Baseline dyspnoea (mMRC) >1	137 (7.68)
Chronic NIV	0 (0)
Home CPAP	0 (0)
Chronic respiratory failure	0 (0)
Comorbidities	
Cardiovascular disease	0 (0)
Chronic pulmonary disease	0 (0)
Ulcerative disease	0 (0)
Liver disease	0 (0)
Nephrological disease	0 (0)
Diabetes	0 (0)
Cancer	0 (0)
Dementia	0 (0)
Cerebrovascular disease	0 (0)
Connective tissue disease	0 (0)
Hemiplegia	0 (0)
AIDS	0 (0)

Arterial hypertension	0 (0)
Charlson Index	0 (0)
Charlson Index>2	0 (0)
Clinical variables in the ED	
Temperature (ºC)	51 (2.86)
HR (bpm)	9 (0.50)
RR (rpm)	508 (28.48)
Mean arterial blood pressure (mmHg)	8 (0.45)
Glasgow	13 (0.73)
APACHE II score in the ED	254 (14.24)
Laboratory variables in the ED	
arterial pCO2 (mmHg)	30 (1.68)
arterial pO2 (mmHg)	81 (4.54)
O2 saturation (%)	48 (2.69)
PaO2/FiO2 (mmHg)	589 (33.02)
Arterial pH	28 (1.57)
Leukocytes (x10^3/mL)	3 (0.17)
Platelets (x10^3/mL)	4 (0.22)
Haematocrit (%)	3 (0.17)
Creatinine (mg/dL)	3 (0.17)
Serum Na+ (mEq/L)	8 (0.45)
Serum K+ (mEq/L)	75 (4.20)
Glucose (mg/dL)	5 (0.28)
Bilirubin (mg/dL)	1017 (57.01)
Albumin (g/dL)	1035 (58.02)
Prothrombin activity (%)	126 (7.06)
Urea (mg/dL)	635 (35.59)
Clinical variables on admission to the RICU	

SBP (mmHg)	3 (0.17)
DBP (mmHg)	3 (0.17)
HR (bpm)	6 (0.34)
RR (rpm)	24 (1.35)
Glasgow	3 (0.17)
APACHE II score on admission	218 (12.22)
Laboratory variables on admission to the RICU	
Arterial pH	14 (0.78)
arterial pCO2 (mmHg)	14 (0.78)
arterial pO2 (mmHg)	29 (1.63)
PaO2/FiO2 (mmHg)	34 (1.91)
Glucose (mg/dL)	13 (0.73)
Albumin (g/dL)	203 (11.38)
Haematocrit (%)	18 (1.01)
ICU admission	0 (0)
Stay in RICU (days)	0 (0)
Hospital stay (days)	0 (0)
In-hospital mortality	0 (0)

The primary study cohort included 1,784 patients. Missing data are reported as number and percentage of missing values for each variable included in the main descriptive tables and in the multivariable analysis. Percentages were calculated using the total primary study cohort as the denominator. AIDS: acquired immunodeficiency syndrome; APACHE II: Acute Physiology and Chronic Health Evaluation II; BMI: body mass index; CPAP: continuous positive airway pressure; DBP: diastolic blood pressure; ED: emergency department; ECOG: Eastern Cooperative Oncology Group performance status; HR: heart rate; ICU: intensive care unit; mMRC: modified Medical Research Council dyspnoea scale; NIV: non-invasive ventilation; PaO_2/FiO_2: ratio of arterial oxygen partial pressure to fraction of inspired oxygen; RICU: respiratory intermediate care unit; RR: respiratory rate; SBP: systolic blood pressure.

Supplementary Table S2A. Baseline socio-demographic characteristics and comorbidities according to time to RICU admission.

	Total (N=1829)	Emergency to RICU group (N=1285)	Admission to the RICU within 24 hours (N=383)	RICU admission between 24 and 48 hours (N=116)	RICU admission between 48 and 72 hours (N=45)	p
Socio-demographic variables						
Sex (male)	1089 (59.54)	785 (61.09)	212 (55.35)	64 (55.17)	28 (62.22)	0.1631
Age (years)	69.35 (14.70)	68.89 (14.79)	69.93 (14.52)	70.96 (14.46)	73.18 (13.87)	0.0362*
Height (m)	1.63 (0.10)	1.63 (0.09)	1.63 (0.10)	1.61 (0.10)	1.62 (0.09)	0.3457
Weight (kg)	78.71 (22.13)	79.11 (21.91)	79.06 (24.01)	71.94 (19.23)	80.36 (14.33)	0.0080*
BMI	29.65 (7.95)	29.76 (7.92)	29.78 (8.54)	27.51 (6.24)	30.76 (6.62)	0.0497*
Baseline situation						
ECOG						0.9787
0, 1	801 (44.70)	572 (45.22)	163 (43.58)	48 (43.24)	18 (42.86)	
2	608 (33.93)	422 (33.36)	134 (35.83)	37 (33.33)	15 (35.71)	
3, 4	383 (21.37)	271 (21.42)	77 (20.59)	26 (23.42)	9 (21.43)	
Baseline dyspnoea (mMRC) >1	952 (56.36)	665 (55.74)	199 (56.70)	59 (57.28)	29 (69.05)	0.3938
Chronic NIV	275 (15.04)	220 (17.12)	46 (12.01)	6 (5.17)	3 (6.67)	0.0004*
Home CPAP	52 (2.84)	41 (3.19)	9 (2.35)	1 (0.86)	1 (2.22)	0.4557
Chronic respiratory failure	620 (33.90)	460 (35.80)	114 (29.77)	29 (25.0)	17 (37.78)	0.0245*
Comorbidities						
Cardiovascular disease	863 (47.18)	588 (45.76)	185 (48.30)	63 (54.31)	27 (60.0)	0.0870
Chronic pulmonary disease	1012 (55.33)	724 (56.34)	202 (52.74)	58 (50.0)	28 (62.22)	0.2875
Ulcerative disease	120 (6.56)	83 (6.46)	26 (6.79)	7 (6.03)	4 (8.89)	0.9179
Liver disease	92 (5.03)	70 (5.45)	15 (3.92)	3 (2.58)	4 (8.89)	0.4025
Nephrological disease	100 (5.46)	72 (5.60)	19 (4.96)	6 (5.18)	3 (6.66)	0.4518
Diabetes	440 (24.06)	313 (24.36)	97 (25.33)	17 (14.66)	13 (28.88)	0.1677
Cancer	142 (7.76)	99 (7.70)	25 (6.53)	13 (11.21)	5 (11.11)	0.3275

Dementia	64 (3.50)	47 (3.66)	14 (3.66)	1 (0.86)	2 (4.44)	0.4520
Cerebrovascular disease	188 (10.28)	125 (9.73)	42 (10.97)	14 (12.07)	7 (15.56)	0.4971
Connective tissue disease	58 (3.17)	41 (3.19)	9 (2.35)	5 (4.31)	3 (6.67)	0.3729
Hemiplegia	46 (2.52)	32 (2.49)	7 (1.83)	7 (6.03)	0	0.0512
AIDS	3 (0.16)	1 (0.08)	1 (0.26)	0	1 (2.22)	0.0055*
Arterial hypertension	1061 (58.01)	744 (57.90)	221 (57.70)	67 (57.76)	29 (64.44)	0.8520
Charlson Index	2 [1-3]	2 [1-3]	1 [1-3]	2 [1-2]	2 [1-4]	0.2082
Charlson Index>2	494 (27.01)	350 (27.24)	97 (25.33)	28 (24.14)	19 (42.22)	0.0957

Data are presented as n (%) unless otherwise stated. Continuous variables are presented as mean (standard deviation), except for the Charlson Comorbidity Index, which is presented as median [interquartile range]. Percentages were calculated using available data for each variable. *$p < 0.05$. AIDS: acquired immunodeficiency syndrome; BMI: body mass index; CPAP: continuous positive airway pressure; ECOG: Eastern Cooperative Oncology Group performance status; mMRC: modified Medical Research Council dyspnoea scale; NIV: non-invasive ventilation; RICU: respiratory intermediate care unit.

This exploratory analysis included patients admitted to the RICU up to 72 hours after hospital admission. The primary analysis cohort included only patients admitted directly from the ED to the RICU or transferred from a conventional ward within 48 hours.

Supplementary Table S2B. Clinical and laboratory variables according to time to RICU admission.

	Total (N=1829)	Emergency to RICU group (N=1285)	Admission to the RICU within 24 hours (N=383)	RICU admission between 24 and 48 hours (N=116)	RICU admission between 48 and 72 hours (N=45)	p
Clinical variables in the ED						
Temperature (ºC)	36.86 (0.82)	36.82 (0.81)	36.95 (0.84)	36.83 (0.79)	37.09 (0.86)	0.0134*
HR (bpm)	100.13 (23.51)	100.94 (23.62)	99.02 (22.97)	96.62 (22.96)	95.36 (25.15)	0.0849
RR (rpm)	22.64 (8.16)	22.81 (8.29)	22.66 (8.21)	21.25 (7.02)	21.19 (6.27)	0.4066
Mean arterial blood pressure (mmHg)	95.10 (20.53)	96.15 (21.11)	93.27 (19.38)	89.85 (18.33)	94.18 (15.58)	0.0039*
Glasgow	14.78 (1.08)	14.77 (1.09)	14.81 (1.03)	14.78 (1.28)	14.95 (0.30)	0.2775
APACHE II score in the ED	15.85 (7.17)	16.04 (7.43)	15.21 (5.81)	15.21 (6.24)	17.76 (10.77)	0.2591
Laboratory variables in the ED						
arterial pCO2 (mmHg)	54.01 (21.93)	55.96 (23.35)	50.48 (18.06)	46.45 (14.72)	46.27 (14.00)	<0.0001*
arterial pO2 (mmHg)	59.39 (22.12)	60.33 (23.76)	57.17 (17.89)	58.40 (14.44)	52.44 (17.57)	0.0321*
O2 saturation (%)	84.60 (11.48)	84.41 (11.85)	84.70 (10.95)	87.12 (8.35)	82.51 (11.15)	0.1618
PaO2/FiO2 (mmHg)	242.42 (91.29)	241.62 (94.60)	246.17 (84.66)	246.58 (78.68)	224.17 (73.90)	0.2600
Arterial pH	7.37 (0.10)	7.36 (0.10)	7.39 (0.08)	7.40 (0.07)	7.42 (0.05)	<0.0001*
Leukocytes ($x10^3$/mL)	11.96 (5.65)	12.14 (5.64)	11.67 (5.51)	11.46 (6.29)	10.48 (5.11)	0.0234*
Platelets ($x10^3$/mL)	233.78 (99.01)	236.41 (101.68)	229.44 (95.87)	221.74 (82.67)	226.16 (83.08)	0.2766
Haematocrit (%)	41.48 (6.67)	41.85 (6.61)	40.60 (6.54)	40.49 (6.75)	40.83 (8.31)	0.0004*
Creatinine (mg/dL)	1.11 (0.71)	1.09 (0.68)	1.16 (0.83)	1.07 (0.60)	1.22 (0.64)	0.2429
Serum Na+ (mEq/L)	138.47 (4.51)	138.66 (4.31)	138.22 (4.71)	137.17 (5.70)	138.20 (4.55)	0.0259*
Serum K+ (mEq/L)	4.58 (0.71)	4.61 (0.71)	4.53 (0.70)	4.49 (0.71)	4.59 (0.76)	0.1375
Glucose (mg/dL)	155.98 (87.10)	157.65 (93.49)	153.61 (72.0)	142.46 (48.52)	162.98 (90.15)	0.6406
Bilirubin (mg/dL)	0.69 (0.53)	0.68 (0.52)	0.62 (0.44)	0.92 (0.81)	0.74 (0.36)	0.0464*
Albumin (g/dL)	3.83 (0.55)	3.85 (0.52)	3.76 (0.65)	3.74 (0.49)	3.86 (0.53)	0.2384
Prothrombin activity (%)	79.34 (26.30)	80.59 (25.63)	76.69 (27.77)	77.18 (26.76)	69.80 (29.19)	0.0027*

Urea (mg/dL)	57.60 (37.37)	56.42 (37.39)	61.28 (38.87)	53.30 (33.62)	68.54 (29.69)	0.0110*
Clinical variables on admission to the RICU						
SBP (mmHg)	129.24 (23.68)	129.61 (23.39)	127.46 (24.01)	129.09 (25.60)	134.18 (23.40)	0.2855
DBP (mmHg)	71.64 (15.53)	72.42 (15.48)	69.61 (15.73)	70.62 (15.71)	69.27 (13.06)	0.0053*
HR (bpm)	91.66 (19.71)	91.05 (19.36)	93.76 (20.47)	93.37 (21.00)	86.93 (18.22)	0.0389*
RR (rpm)	22.82 (6.36)	22.41 (6.20)	23.27 (6.16)	25.29 (7.72)	24.22 (7.08)	0.0004*
Glasgow	14.69 (1.29)	14.75 (1.09)	14.57 (1.67)	14.46 (1.80)	14.62 (1.27)	0.0236*
APACHE II score on admission	12.24 (5.65)	11.89 (5.48)	12.96 (5.97)	13.31 (6.13)	13.63 (5.82)	0.0032*
Laboratory variables on admission to the RICU						
Arterial pH	7.39 (0.08)	7.38 (0.07)	7.38 (0.08)	7.38 (0.09)	7.37 (0.08)	0.1894
arterial pCO2 (mmHg)	52.06 (19.15)	51.12 (18.41)	53.47 (20.23)	55.57 (21.41)	58.09 (22.21)	0.0326*
arterial pO2 (mmHg)	73.00 (27.98)	74.77 (30.58)	69.05 (20.27)	70.56 (19.66)	61.86 (15.34)	0.0002*
PaO2/FiO2 (mmHg)	230.59 (92.21)	235.86 (93.91)	224.31 (87.33)	211.70 (85.96)	179.30 (73.92)	0.0001*
Glucose (mg/dL)	159.11 (68.72)	154.98 (63.67)	170.19 (82.04)	164.50 (66.98)	170.24 (78.78)	0.0114*
Albumin (g/dL)	3.78 (0.57)	3.80 (0.56)	3.73 (0.58)	3.70 (0.57)	3.68 (0.56)	0.0060*
Haematocrit (%)	39.74 (6.80)	40.07 (6.78)	39.12 (6.62)	38.80 (7.12)	37.70 (7.37)	0.0050*

Data are presented as mean (standard deviation). Values were calculated using available data for each variable. *$p < 0.05$. APACHE II: Acute Physiology and Chronic Health Evaluation II; DBP: diastolic blood pressure; ED: emergency department; HR: heart rate; PaO_2/FiO_2: ratio of arterial oxygen partial pressure to fraction of inspired oxygen; RICU: respiratory intermediate care unit; RR: respiratory rate; SBP: systolic blood pressure.

This exploratory analysis included patients admitted to the RICU up to 72 hours after hospital admission. The primary analysis cohort included only patients admitted directly from the ED to the RICU or transferred from a conventional ward within 48 hours.

Supplementary Table S2C. Primary diagnosis according to time to RICU admission.

	Total (N=1829)	Emergency to RICU group (N=1285)	Admission to the RICU within 24 hours (N=383)	RICU admission between 24 and 48 hours (N=116)	RICU admission between 48 and 72 hours (N=45)	p
COPD	407 (22.25)	318 (24.75)	68 (17.75)	17 (14.66)	4 (8.89)	0.0005*
OHS	160 (8.75)	110 (8.56)	38 (9.92)	7 (6.03)	5 (11.11)	0.5514
Heart failure	211 (11.54)	134 (10.43)	55 (14.36)	18 (15.52)	4 (8.89)	0.0839
Pneumonia	372 (20.34)	248 (19.30)	91 (23.76)	24 (20.69)	9 (20.00)	0.3037
Pulmonary embolism	330 (18.04)	257 (20.00)	53 (13.84)	18 (15.52)	2 (4.44)	0.0029*
Asthma	88 (4.81)	61 (4.75)	20 (5.22)	4 (3.45)	3 (6.67)	0.8106
ILD	45 (2.46)	20 (1.56)	9 (2.35)	6 (5.17)	10 (22.22)	<0.0001*
Neuromuscular disease	26 (1.42)	17 (1.32)	6 (1.57)	3 (2.59)	0	0.5895
Other diagnoses	190 (10.39)	120 (9.34)	43 (11.23)	19 (16.38)	8 (17.78)	0.0303*

Data are presented as n (%). Percentages were calculated using the total number of patients in each admission-time category as the denominator. For each diagnostic category, the p value refers to the comparison between patients with that diagnosis and patients with all other primary diagnoses across admission-time categories. *$p < 0.05$. COPD: chronic obstructive pulmonary disease; ILD: interstitial lung disease; OHS: obesity hypoventilation syndrome; RICU: respiratory intermediate care unit. The "Other diagnoses" category included other restrictive respiratory disorders and less frequent respiratory conditions not captured by the predefined diagnostic categories.

This exploratory analysis included patients admitted to the RICU up to 72 hours after hospital admission. The primary analysis cohort included only patients admitted directly from the ED to the RICU or transferred from a conventional ward within 48 hours.

Supplementary Table S2D. Clinical outcomes according to time to RICU admission.

	Total (N=1829)	Emergency to RICU group (N=1285)	Admission to the RICU within 24 hours (N=383)	RICU admission between 24 and 48 hours (N=116)	RICU admission between 48 and 72 hours (N=45)	p
ICU admission	67 (3.66)	33 (2.57)	21 (5.48)	9 (7.76)	4 (8.89)	0.0007*
RICU length of stay (days)	3 [2-5]	3 [2-5]	3 [2-5]	3 [2-5]	4 [2-6]	0.1428
Hospital length of stay (days) *	6 [4-8]	6 [4-8]	6 [5-9]	6 [4-9]	7 [4-13]	0.0038*
In-hospital mortality	156 (8.53)	74 (5.76)	43 (11.23)	24 (20.69)	15 (33.33)	<0.0001*

Data are presented as n (%) unless otherwise stated. Length-of-stay variables are presented as median [interquartile range]. *$p < 0.05$. ICU: intensive care unit; RICU: respiratory intermediate care unit.

This exploratory analysis included patients admitted to the RICU up to 72 hours after hospital admission. The primary analysis cohort included only patients admitted directly from the ED to the RICU or transferred from a conventional ward within 48 hours.

Supplementary Table S3. Comparative analysis of the primary study cohort according to admission period (N = 1784).

Variables	Admission period			p
	2007-2009 (N=511)	2010-2012 (N=537)	2013-2016 (N=736)	
Admission pathway				0.9678
Direct RICU admission	366 (71.62)	387 (72.07)	532 (72.28)	
Early ward-to-RICU transfer	145 (28.38)	150 (27.93)	204 (27.72)	
Socio-demographic variables				
Sex (male)	322 (63.01)	320 (59.59)	419 (56.93)	0.0984
Age (years)	68.14 (14.75)	70.02 (13.90)	69.46 (15.23)	0.0539
Height (m)	1.63 (0.10)	1.63 (0.11)	1.63 (0.09)	0.8981
Weight (kg)	80.94 (24.06)	76.85 (21.17)	78.77 (21.97)	0.0559
BMI	30.68 (8.67)	28.81 (7.34)	29.65 (7.98)	0.0125*
Baseline situation				
ECOG				0.0971
0, 1	240 (49.28)	223 (42.00)	320 (43.72)	
2	152 (31.21)	180 (33.90)	261 (35.66)	
3, 4	95 (19.51)	128 (24.11)	151 (20.63)	
Baseline dyspnoea (mMRC) >1	254 (53.59)	293 (58.60)	376 (55.87)	0.2870
Chronic NIV	83 (16.24)	81 (15.08)	108 (14.67)	0.7445
Home CPAP	9 (1.76)	13 (2.42)	29 (3.94)	0.0582
Chronic respiratory failure	181 (35.42)	183 (34.08)	239 (32.47)	0.5494
Charlson Index	2 [1-3]	2 [1-3]	1 [0-2]	<0.0001*
Charlson Index>2	158 (30.95)	158 (29.42)	159 (21.60)	0.0003*
APACHE II score in the ED	16.83 (7.22)	16.11 (6.53)	14.89 (7.19)	<.0001*
APACHE II score on admission to the RICU	13.19 (5.92)	12.77 (5.80)	11.12 (5.14)	<.0001*
Primary diagnosis				

COPD	120 (23.48)	108 (20.11)	175 (23.78)	0.2576
OHS	64 (12.52)	38 (7.08)	53 (7.20)	0.0013*
Heart failure	41 (8.02)	78 (14.53)	88 (11.96)	0.0042*
Pneumonia	113 (22.11)	110 (20.48)	140 (19.02)	0.4091
Pulmonary embolism	76 (14.87)	97 (18.06)	155 (21.06)	0.0208*
Asthma	27 (5.28)	24 (4.47)	34 (4.62)	0.8022
ILD	8 (1.57)	10 (1.86)	17 (2.31)	0.6350
Neuromuscular disease	4 (0.78)	10 (1.86)	12 (1.63)	0.3034
Other diagnoses	58 (11.35)	62 (11.55)	62 (8.42)	0.1145
Clinical outcomes				
ICU admission	21 (4.11)	21 (3.91)	21 (2.85)	0.4228
RICU length of stay (days)	3 [2-5]	3 [2-5]	3 [2-5]	0.0794
Hospital length of stay (days)	6 [4-8]	6 [4-8]	6 [4-8]	0.4089
In-hospital mortality	50 (9.78)	49 (9.12)	42 (5.71)	0.0145*

Data are presented as n (%) unless otherwise stated. Continuous variables are presented as mean (standard deviation), except for the Charlson Comorbidity Index, RICU length of stay, and hospital length of stay, which are presented as median [interquartile range]. Percentages were calculated using available data for each variable. For each diagnostic category, the p value refers to the comparison between patients with that diagnosis and patients with all other primary diagnoses across admission periods. *$p < 0.05$. APACHE II: Acute Physiology and Chronic Health Evaluation II; BMI: body mass index; COPD: chronic obstructive pulmonary disease; CPAP: continuous positive airway pressure; ECOG: Eastern Cooperative Oncology Group performance status; ICU: intensive care unit; ILD: interstitial lung disease; mMRC: modified Medical Research Council dyspnoea scale; NIV: non-invasive ventilation; OHS: obesity hypoventilation syndrome; RICU: respiratory intermediate care unit.

Supplementary Table S4. Standardized mean differences for variables included in the multivariable model in the complete-case cohort.

Variables	Direct RICU admission group (N=1094)	Early ward-to-RICU transfer group (N=408)	SMD
Sex			
Female	442 (40.4)	189 (46.3)	0.120
ECOG			0.032
0, 1	509 (46.5)	185 (45.3)	
2	360 (32.9)	134 (32.8)	
3, 4	225 (20.6)	89 (21.8)	
APACHE II, Emergency Department	16.06 (7.43)	15.11 (5.89)	0.142
Charlson index	1.74 (1.55)	1.68 (1.56)	0.038
Diagnostics (Binary variables)			
Heart failure	112 (10.2)	57 (14.0)	0.115
ILD	17 (1.7)	13 (3.2)	0.107

Data are presented as n (%) unless otherwise stated. Standardized mean differences were calculated for variables included in the multivariable model in the complete-case cohort. Values <0.10 were considered to indicate good balance, whereas values >0.10 suggested small between-group imbalance. APACHE II: Acute Physiology and Chronic Health Evaluation II; ECOG: Eastern Cooperative Oncology Group performance status; ILD: interstitial lung disease; RICU: respiratory intermediate care unit; SMD: standardized mean difference.
